# Susceptibility of Influenza A, B, C, and D Viruses to Baloxavir^1^

**DOI:** 10.3201/eid2510.190607

**Published:** 2019-10

**Authors:** Vasiliy P. Mishin, Mira C. Patel, Anton Chesnokov, Juan De La Cruz, Ha T. Nguyen, Lori Lollis, Erin Hodges, Yunho Jang, John Barnes, Timothy Uyeki, Charles T. Davis, David E. Wentworth, Larisa V. Gubareva

**Affiliations:** Centers for Disease Control and Prevention, Atlanta, Georgia, USA (V.P. Mishin, M.C. Patel, A. Chesnokov, J. De La Cruz, H.T. Nguyen, L. Lollis, E. Hodges, Y. Jang, J. Barnes, T. Uyeki, C.T. Davis, D.E. Wentworth, L.V. Gubareva);; Battelle Memorial Institute, Atlanta (M.C. Patel, J. De La Cruz, H.T. Nguyen, L. Lollis)

**Keywords:** baloxavir, baloxavir marboxil, drug susceptibility, zoonotic influenza, avian influenza, influenza viruses, viruses, H7N9, pandemic potential, highly pathogenic viruses, respiratory infections, zoonoses, United States

## Abstract

Baloxavir showed broad-spectrum in vitro replication inhibition of 4 types of influenza viruses (90% effective concentration range 1.2–98.3 nmol/L); susceptibility pattern was influenza A ˃ B ˃ C ˃ D. This drug also inhibited influenza A viruses of avian and swine origin, including viruses that have pandemic potential and those resistant to neuraminidase inhibitors.

Influenza viruses are classified into 4 types: A, B, C, and D (*1*). Influenza A viruses infect a wide range of species and pose threats to human and animal health. Influenza A viruses belonging to 16 hemagglutinin and 9 neuraminidase subtypes have been identified in the natural reservoir (wild birds). Zoonotic infections with avian H5N1, H5N6, and H7N9 viruses are concerning because of their high fatality rates in humans and pandemic risk (*2*).

Swine are recognized as mixing vessels because influenza A viruses from multiple hosts can infect pigs and produce novel reassortants. Numerous subtypes of reassortant swine influenza A viruses are enzootic throughout North America and pose a threat to human health. For instance, H3N2 triple reassortant viruses caused a multistate outbreak affecting hundreds of persons in the United States during 2012, and a quadruple reassortant H1N1 virus caused the 2009 pandemic and now circulates as a seasonal virus (*2*,*3*).

Influenza B viruses are considered strictly human pathogens, although occasional outbreaks in aquatic mammals have been reported (*1*). Influenza C viruses are known to infect humans, pigs, camels, and dogs (*1*). Unlike influenza A and B viruses, influenza C viruses typically cause mild illness. However, in recent years, severe illness in children infected by influenza C virus has raised concerns over the lack of virus-specific therapeutics and vaccines (*4*). Recently discovered influenza D viruses were isolated from swine and bovines. No virologically confirmed human infections have been reported, but influenza D virus antibodies have been found in persons exposed to cattle (*1*). Evolutionarily, influenza C and D viruses are more closely related to each other than to influenza A or B viruses (*1*).

Antiviral drugs have been used to mitigate zoonotic virus outbreaks and are central to pandemic preparedness. However, therapeutic options remain limited and drug-resistant viruses can emerge after treatment, spontaneous mutation, or reassortment. Until recently, only matrix (M) 2 blockers and neuraminidase inhibitors (NAIs) were approved to control influenza. M2 blockers are effective only against influenza A viruses and are not recommended because of widespread resistance. NAIs are used for treatment of influenza A and B virus infections, but NAI-resistant viruses have emerged (*5*). NAI-resistant seasonal influenza H1N1 viruses circulated worldwide during late 2007 through early 2009 (*6*) and raised concerns over limited therapeutic options.

In 2014, favipiravir was licensed in Japan for restricted use in the event of a drug-resistant influenza pandemic (*7*). Favipiravir is a broad-spectrum antiviral drug that inhibits viral RNA polymerase, an enzyme recognized as an attractive target because of its critical role in virus replication and high degree of conservation (*8*). In 2018, another inhibitor of the viral RNA polymerase, baloxavir marboxil, was approved in Japan and the United States for treatment of influenza A and B virus infections (*9*). Its active metabolite, baloxavir acid, inhibits cap-dependent endonuclease activity of polymerase acidic (PA) protein (*10*). Amino acid substitutions at position 38 in the PA active site were recognized as the primary pathway to baloxavir resistance (*11*). PA substitutions at this and other positions have variable impact on resistance and are rarely found in nature (*11*,*12*). The purpose of this study was to determine the effectiveness of baloxavir against the 4 types of influenza viruses.

## The Study

The active site of the PA protein (P3 in C and D viruses) is nearly identical in all 4 influenza virus types (*1*,*8*). Therefore, we hypothesized that baloxavir would inhibit replication of not only influenza A and B viruses but also influenza C and D viruses. First, we tested 2 viruses of each type by using a virus yield reduction assay. We used baloxavir acid (baloxavir) in experiments and included favipiravir as a control.

Baloxavir broadly inhibited virus replication of all 4 types (Table 1). On the basis of 90% effective concentration values determined at 48 hours postinfection, influenza A viruses were most susceptible to baloxavir and influenza D viruses least susceptible. Baloxavir susceptibility for influenza B viruses was ≈3-fold lower and that for influenza C viruses was ≈6-fold lower than that for influenza A viruses. Analysis of 34 P3 sequences of influenza D virus and 221 of influenza C virus (retrieved from GISAID, https://www.gisaid.org, and GenBank) showed that all influenza D viruses have valine at position 38, whereas influenza C viruses have isoleucine, similar to most influenza A and B viruses. Nevertheless, valine at 38 in influenza A and B viruses had little or no effect (<3-fold) on baloxavir susceptibility (*10**–**12*). Favipiravir also showed inhibitory effects against all virus types, although much higher concentrations were required to achieve similar levels of reduction (Table 1).

**Table 1:** Drug susceptibility of influenza A, B, C, and D viruses by viral yield reduction assay in MDCK cells*

Type	Virus	Virus titer, log_10_ TCID_50_/mL†			EC_90_, nmol/L			
					Baloxavir, mean ± SD			Favipiravir
		24 hpi	48 hpi		24 hpi	48 hpi		48 hpi
A	A/Texas/138/2018 (H1N1)pdm09	6.4 ± 0.5	9.1 ± 0.1		0.8 ± 0.2	1.2 ± 0.1		NT
	A/Illinois/08/2018 (H1N1)pdm09	5.8 ± 0.5	8.9 ± 0.3		2.7 ± 0.5	3.3 ± 0.2		3,005
B	B/Maryland/29/2018	3.5 ± 0.5	7.1 ± 0.6		8.9 ± 1.6	5.8 ± 1.1		1,789
	B/Iowa/18/2018	3.5 ± 0.5	7.6 ± 0.4		13.8 ± 2.0	7.8 ± 1.7		1,635
C	C/Taylor/1233/47	<1.5	5.9 ± 0.5		NA	13.0 ± 3.3		27,476
	C/Aomori/74	<1.5	4.9 ± 0.4		NA	18.4 ± 6.5		31,603
D	D/swine/Oklahoma/13334/2011	4.4 ± 0.3	7.7 ± 0.1		110.2 ± 27.6	98.3 ± 23.9		2,764
	D/bovine/Oklahoma/660/2013	4.8 ± 0.0	8.0 ± 0.5		105.6 ± 37.0	64.3 ± 16.2		3,106

*Cell monolayers were inoculated at a multiplicity of infection of 0.0005 and virus was allowed to adsorb for 1 h. Virus inoculum was removed, serially diluted drug (baloxavir: 0.5–500 nmol/L; favipiravir: 310–318,000 nmol/L) was added, and cells were incubated at 33°C in a 5% CO_2_ incubator. At 24 and 48 hpi, cell culture supernatants were harvested to determine infectious virus titers. Replication of influenza A and B viruses was detected by neuraminidase activity (Fluor-NA Kit; Applied Biosystems, https://www.thermofisher.com), and replication of influenza C and D viruses by esterase activity (3-acetyl-umbelliferone in 20 mmol/L Tris-HCl, pH 8.0, reaction buffer (Sigma-Aldrich, https://www.sigmaaldrich.com). The EC_90_ corresponds to a drug concentration causing a 90% reduction in virus titer compared with control wells without drug. The EC_90_ for each virus and drug were determined by using nonlinear regression analysis (GraphPad, https://www.graphpad.com). For baloxavir, results are shown as mean ± SD of 3 independent experiments; favipiravir results are shown as single or average of 2 independent experiments. EC_90_, 90% effective concentration; hpi, hours postinfection; NA, not applicable; NT, not tested; TCID_50_, 50% tissue culture infectious dose. †Virus titers were determined in control wells without drug.

Although the virus yield reduction assay has been used to assess baloxavir susceptibility of seasonal and avian viruses (*10*,*13*), other phenotypic assays, such as the focus-reduction assay (FRA) and the high-content imaging neutralization test (HINT), offer an improved throughput (*12*,*14*,*15*). Regardless of the assay used, baloxavir effective concentrations for influenza A viruses were similar (≈0.1–3 nmol/L) (*10*,*12*–*15*). Unlike the FRA, HINT relies on single-cycle virus replication, which is achieved by withdrawing trypsin needed to activate infectivity of progeny virus. HINT eliminates variance caused by different replication kinetics. However, the FRA is optimal for testing highly pathogenic avian viruses because multicycle replication of these viruses is trypsin independent. We used 2 seasonal A(H1N1)pdm09 viruses, one of which contains the naturally occurring substitution PA-I38L, for reference purposes (*12*) (Table 2).

**Table 2:** Baloxavir susceptibility of zoonotic and animal influenza A viruses in MDCK-SIAT1 cells*

Virus subtype	FRA			HINT	
	No. viruses tested	EC_50_, nmol/L, mean ± SD†		No. viruses tested	EC_50_, nmol/L, mean ± SD†
Avian origin					
H5N6‡	3	0.31 ± 0.19		–	–
H6N1	1	0.12		1	0.48
H7N9	19	0.48 ± 0.32		9	1.44 ± 1.08
H9N2§	1	0.18		1	0.53
H10N8	1	0.30		1	0.63
Swine origin					
H1N1¶	–	–		3	0.72 ± 0.27
H1N1v	–	–		3	0.51 ± 0.12
H1N2v	–	–		9	1.19 ± 0.36
H3N2v	–	–		15	0.86 ± 0.50
Reference viruses#					
A/Illinois/08/2018 (H1N1)pdm09 PA-I38		2.12		–	1.75 ± 0.59
A/Illinois/37/2018 (H1N1)pdm09 PA-I38L		14.96 (7-fold)**		–	13.09 ± 3.56 (8-fold)**

*Both assays were conducted by using MDCK-SIAT1 cells. Details on viruses tested are in the Appendix. According to World Health Organization nomenclature, the swine-origin influenza viruses isolated from humans are named variant viruses (e.g., A[H1N1]v). EC_50_, 50% effective concentration; FRA, focus reduction assay; HINT, high-content imaging neutralization test; –, not tested. †Mean ± SD or average of 2 test results. ‡One of 3 viruses was isolated from chicken (Appendix Table 1). §Virus was isolated from chicken (Appendix Table 1). ¶All 3 viruses were isolated from swine (Appendix Table 2). #A pair of seasonal influenza A(H1N1)pdm09 viruses were included in each test as reference viruses (*12*). **Fold change to EC_50_ of virus carrying PA-I38L compared with sequence-matched control virus carrying PA-I38.

First, we tested 25 influenza viruses of avian origin, representing H5, H6, H7, H9, and H10 subtypes, by using FRA or HINT as described (*12*) (Table 2; Appendix Table 1). Most viruses were isolated from infected humans. Most viruses had markers of M2 resistance and some had NAI-resistance markers. Data showed that these diverse viruses were susceptible to baloxavir and had 50% effective concentration (EC_50_) values in a low nanomolar range (Table 2; Appendix Table 1). In the FRA, favipiravir EC_50_ values were much higher than those for baloxavir (Appendix Table 1). However, favipiravir did not produce a measurable antiviral effect by HINT because this drug requires several hours for activation in cells. Baloxavir susceptibility of 30 swine-origin viruses, representing different lineages and subtypes and collected over many years, demonstrated HINT EC_50_ values comparable to avian and seasonal influenza A viruses (Table 2; Appendix Table 2) (*10*,*12**,**13*).

It is prudent to analyze PA sequences of emerging influenza A viruses for markers previously associated with reduced baloxavir susceptibility (*11*,*12*). Among swine-origin viruses available for testing in this study, polymorphism PA-38I/M was detected in A/Iowa/33/2017 (H1N1)v. Virus populations with either PA-I38 or PA-I38M were recovered by biologic cloning and tested by using HINT. Substitution PA-I38M conferred 12-fold reduced baloxavir susceptibility, consistent with previous reports for PA-I38M–containing H3N2 viruses (*11*,*12*). Analysis of PA sequences from 2,485 H7N9 viruses (from GISAID and GenBank) showed 1 virus with PA-I38M, 2 with PA-E199G, and 1 with PA-A36V (*11*,*12*). The effect of these substitutions on baloxavir susceptibility for H7N9 viruses is currently unknown. Moreover, PA sequence of 1 swine influenza A virus showed PA-I38T, a marker associated with clinically relevant baloxavir resistance (*11*). None of these viruses were available for phenotypic testing.

## Conclusions

Baloxavir displayed broad antiviral activity against diverse influenza viruses, including all 4 types and animal-origin influenza A viruses with pandemic potential. Our findings suggest that baloxavir might offer the first therapeutic option against influenza C virus infections. Further studies are needed to provide comprehensive assessment of baloxavir susceptibility by using a large panel of representative influenza C viruses. Ongoing monitoring of baloxavir susceptibility of emerging avian and swine influenza A viruses with pandemic potential is needed to inform clinical management and public health preparedness efforts.

Appendix Additional information on susceptibility of influenza A, B, C, and D viruses to baloxavir.
