# Supplementary material for: Susceptibility of Influenza A, B, C, and D Viruses to Baloxavir
Source: Emerg Infect Dis. 2019 Oct;25(10):1969–72. doi: 10.3201/eid2510.190607 (PMC6759234; doi:10.3201/eid2510.190607)
Supplement: Appendix — Additional information on susceptibility of influenza A, B, C, and D viruses to baloxavir. [file 19-0607-Techapp-s1.pdf]

Article DOI: https://doi.org/10.3201/eid2510/190607

Susceptibility of Influenza A, B, C, and D Viruses to Baloxavir

Appendix.

Appendix Table 1. Drug susceptibility of zoonotic and avian influenza A viruses*

Subtype	Virus name	M2 blocker resistance marker in M2 protein	NAI resistance marker in NA protein	PA gene accession no.	FRA: Favipiravir, EC_{50}, nmol/L†	FRA: Baloxavir, EC_{50}, nmol/L†	HINT: Baloxavir, EC_{50}, nmol/L†
H5N6	A/Sichuan/26221/2014‡	None	None	EPI533587	–	0.11, 0.22	–
	A/Yunnan/14563/2015‡	S31N	None	EPI587617	–	0.58, 0.56	–
	A/chicken/Vietnam/NCVD-16A26/2016‡	–	None	EPI1335786	39,530, 48,270	0.17, 0.19	–
H6N1	A/Taiwan/2/2013	S31N	None	EPI459854	33,620, 33,180	0.10, 0.14	0.38, 0.57
H7N9	A/Anhui/1/2013	S31N	None	EPI439508	–	0.39, 0.41	1.03, 1.28
(wave 1)	A/Shanghai/1/2013	S31N	R292K	EPI439490	28,800	0.40, 0.42	0.99, 1.29
	A/Shanghai/2/2013	S31N	None	EPI439498	–	0.29, 0.33	–
	A/Taiwan/1/2013 clone 1	S31N	None	EPI515451	–	1.04, 0.85	0.63, 0.68
	A/Taiwan/1/2013 clone 2	S31N	E119V	EPI515458	26,940	0.92, 1.30	0.66, 0.56
	A/Taiwan/1/2013 clone 3	S31N	I222K	EPI515474	–	0.66, 0.58	1.71, 1.34
	A/Taiwan/1/2013 clone 4	S31N	R292K	EPI516361	18,820	0.62, 0.50	0.62, 0.56
	A/Taiwan/1/2013 clone 5	S31N	I222R	EPI515466	–	0.35, 0.46	0.78, 0.87
H7N9	A/Hong Kong/5942/2013	S31N	None	EPI490878	–	0.47, 0.43	–
(wave 2)	A/Hong Kong/2212982/2014	S31N	None	EPI502369	–	0.05, 0.07	–
	A/Hong Kong/734/2014	S31N	None	EPI498796	–	0.26, 0.21	–
H7N9	A/Hong Kong/56/2015	S31N	None	EPI1489673	–	0.11, 0.15	–
(wave 3)	A/British Columbia/1/2015	S31N	None	EPI560397	–	0.15, 0.19	–
H7N9 (wave 4)	A/Hong Kong/793/2016	S31N	None	Pending	–	0.72, 1.01	–
H7N9	A/Hong Kong/61/2016	S31N	None	EPI1335764	27,390	0.46, 0.33	2.39, 2.36
(wave 5)	A/Hong Kong/4553/2016	S31N	None	EPI1335756	44,250, 34,200	0.68, 0.92	3.98, 4.18
	A/Hong Kong/125/2017	S31N	None	EPI977391	–	0.18, 0.17	–
	A/Hong Kong/214/2017	S31N	None	EPI884218	–	0.07, 0.07	–
	A/Taiwan/1/2017‡	S31N	R292K	EPI917064	56,240, 53,170	0.95, 1.08	–
H9N2	A/chicken/Vietnam/NCVD-LS52/2016	S31N	None	EPI1335772	20,120, 20,870	0.17, 0.18	0.63, 0.43
H10N8	A/Jiangxi/09037/2014	S31N	None	EPI530446	34,000, 32,980	0.26, 0.34	0.81, 0.45

*All procedures were conducted in Biosafety Level 3–enhanced containment. Both assays were conducted by using MDCK-SIAT1 cells. Most viruses were tested in duplicate to determine EC_{50}. EC_{50}, 50% effective concentration; FRA, focus reduction assay; HINT, high-content imaging neutralization test; M2, matrix 2; NA, neuraminidase; NAI, neuraminidase inhibitor; PA, polymerase acidic; –, not tested.
†Duplicate EC_{50} values for each virus are shown.
‡Highly pathogenic avian influenza virus.

Appendix Table 2. Baloxavir susceptibility of swine and variant influenza A viruses determined by HINT*

Subtype	Virus name	M2 blocker resistance marker in M2 protein	PA gene accession no.	Baloxavir EC_{50}, nmol/L†
H1N1	A/swine/Iowa/15/30	None	EPI129822	0.45, 1.05
	A/swine/1976/31	–	–	1.03, 1.06
	A/swine/Tennessee/1/75	None	EPI62684	0.42, 0.33
H1N1v	A/South Dakota/03/2008	V27T	EPI291878	0.45, 0.48
	A/Texas/14/2008	V27T	EPI291886	0.62, 0.73
	A/Minnesota/33/2014	S31N	EPI558110	0.40, 0.40
H1N2v	A/Michigan/09/2007	None	EPI291823	1.39 ± 0.15
	A/Ohio/24/2017	S31N	EPI1056721	0.53 ± 0.14
	A/Ohio/35/2017	S31N	EPI1056729	1.30, 1.20
	A/California/62/2018	S31N	EPI1311357	1.80, 1.90
	A/California/63/2018	S31N	EPI1311365	1.40, 1.30
	A/Michigan/382/2018	S31N	EPI1271030	0.80, 1.10
	A/Michigan/383/2018	S31N	EPI1271062	0.80, 0.90
	A/Michigan/384/2018	S31N	EPI1271038	1.60, 1.20
	A/Ohio/25/2018	S31N	EPI1311349	1.30, 1.00
H3N2v	A/Ohio/88/2012‡	S31N	EPI541959	1.15; 0.95
	A/Iowa/04/2013	S31N	EPI482821	1.34, 2.30
	A/Ohio/4319/2014	S31N	EPI397958	0.90 ± 0.17
	A/Wisconsin/24/2014	S31N	EPI557538	1.46 ± 0.46
	A/Ohio/02/2014	S31N	EPI539158	0.46 ± 0.14
	A/Michigan/39/2015	S31N	EPI642509	0.36, 0.78
	A/Michigan/83/2016	S31N	EPI824771	1.60, 1.60
	A/Michigan/84/2016	S31N	EPI838247	1.00 ± 0.12
	A/Ohio/28/2016	S31N	EPI824753	0.10, 0.21
	A/Ohio/27/2016	S31N	EPI824745	0.58, 0.62
	A/North Dakota/19/2017	S31N	EPI1311461	0.32, 1.05
	A/Ohio/14/2017	S31N	EPI1056657	0.06, 0.10
	A/Ohio/15/2017	S31N	EPI1056665	1.19, 1.23
	A/Ohio/29/2017	S31N	EPI1311405	0.29 ± 0.12
	A/Ohio/13/2017	S31N	EPI1056649	0.70, 1.19

*HINT was conducted by using MDCK-SIAT1 cells. All procedures involving swine-origin viruses were conducted in Biosafety Level 2–enhanced containment. EC_{50}, 50% effective concentration; HINT, high-content imaging neutralization test; M2, matrix ; PA, polymerase acidic; –, not tested.
†EC_{50} values from 2 tests. Mean ± SD values are shown for viruses which were tested more than twice.
‡Contains NA-S247P, a marker associated with decreased susceptibility to neuraminidase inhibitors.
